# A Rare Form of* Brucella* Bursitis with Negative Serology: A Case Report and Literature Review

**DOI:** 10.1155/2017/9802532

**Published:** 2017-02-26

**Authors:** F. M. Almajid

**Affiliations:** Infectious Diseases Unit, Department of Medicine, College of Medical Sciences, King Saud University, Riyadh, Saudi Arabia

## Abstract

Brucellosis is still endemic in certain parts of the world including the Mediterranean, the Middle East, Latin America, and African regions. Osteoarticular manifestations are common presenting features. Brucellosis presenting as prepatellar bursitis has already been reported. We present a case of seronegative olecranon bursitis with positive blood and aspirate cultures. The patient improved remarkably by treatment with streptomycin and doxycycline with no evidence or relapse.

## 1. Introduction

Brucellosis is a zoonotic infection caused by* Brucella* spp. [1] and is prevalent all over the world. The Kingdom of Saudi Arabia still remains an endemic area with national annual incidence of 137.61 per 100,000 [2]. The disease is transmitted to humans by consumption of unpasteurized dairy products or direct contact with infected animals, placentas, or aborted fetuses. It runs an insidious course with varying degrees of complications. Auxiliary arthritis, spondylitis, and sacroiliitis are common manifestations of brucellosis. The incidence of osteoarticular manifestations ranges between 10 and 80% with the majority of reports documenting it between 20 and 40 per cent [3]. Brucellosis presenting as bursitis only is rare as it is usually accompanied by other joint involvement and accounts for just 1–7% of bone and joint disease [4, 5]. Around 50 cases of* Brucella* bursitis involving different sites have been reported in the literature. Apart from 2 cases from Australia and UK [6], all reported cases originated from endemic countries of brucellosis. Prepatellar bursa is the most common site of* Brucella* infection according to all cases published in English literature (Table 1). The diagnosis of brucellosis is often challenging in view of the insidious nature of the illness, slow growth rate of the organism in culture media, and the complexity and unpredictability of most of the established serologic tests [7]

This unusual clinical site and laboratory findings of this patient with* Brucella* bursitis is presented.

I described in this case report the first case of* Brucella* bursitis with negative serology before the diagnosis been identified by aspirate and blood culture. I hope that clinicians will consider brucellosis in both acute and chronic bursitis especially in endemic areas and send bursal fluid and blood culture.

## 2. Case History

A 43-year-old Saudi male suffering from diabetes and hypertension presented in infectious diseases clinic with four months' history of painful swollen joints. At the onset the patient noticed a painless swelling over his elbow. After a month he developed progressive pain and swelling in his right ankle and right thumb over a period of three months. Furthermore, he developed lower back pain but denied having fever. He admitted consumption of raw milk regularly, in addition to prolonged history of contact with camels. There was no significant history of travel. He denied using intravenous drug or alcohol intake. Physical examination revealed an apparently healthy man, afebrile, with normal chest and cardiovascular system.

### 2.1. Examinations

**Figure 1:**
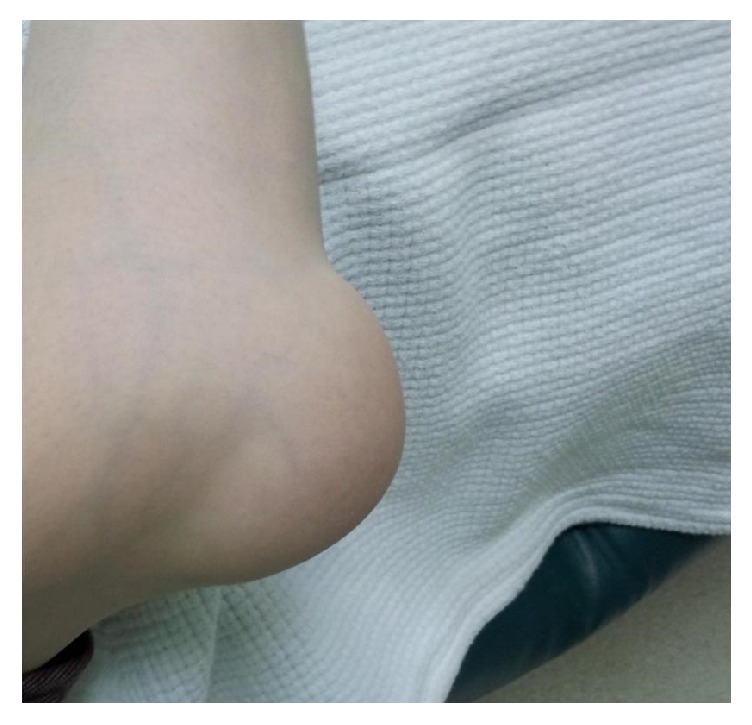
Showing swelling around the right elbow representing olecranon bursitis.

Musculoskeletal examination revealed presence of large soft tissue swelling over the elbow joint with minimal signs of inflammation (Figure 1). There was also swelling of the right ankle with clinical evidence of inflammation. In addition to tenderness and synovial thickening involving the right thumb joint, patient was diagnosed to have right olecranon bursitis and arthritis involving the right ankle and right thumb. Laboratory parameters revealed white blood cells of 9 × 10^9^/L, hemoglobin: 14 g/dl, platelets of 180,000/mcL, and erythrocyte sedimentation rate (ESR) 120 mm/hr. Urea, electrolytes, and liver function tests were within normal limits. Antinuclear antibody test and rheumatoid factor were negative. Patient was diagnosed to have seronegative rheumatoid arthritis and treated with nonsteroidal anti-inflammatory drugs along with local steroid injection of the bursa. Brucella serology tested negative by standard tube agglutination test, repeated at 2-week interval in order to overcome the problem of premature serology test and Coombs test was done to detect the presence of non-agglutinating antibodies for brucellosis. After 72 h incubation, colonies of a small Gram-negative coccobacillus were noted on the blood and chocolate agar plates from both samples, blood and olecranon bursa aspirates. The* Brucella* spp. was sensitive to tetracycline, cotrimoxazole, streptomycin, and rifampicin. The patient was treated with streptomycin 1 g intramuscularly once daily for 2 weeks and doxycycline 100 mg orally twice daily for 3 months. Patient recovered and was followed up for 2 years with no evidence of relapse.

## 3. Discussion

Osteoarticular disease is among common complications of brucellosis. In the Kingdom of Saudi Arabia 47.7% of patients with brucellosis present with osteoarticular manifestations with a substantial number of patients presenting with peripheral arthritis, sacroiliitis, and destructive spondylitis [8]. Bursitis is considered to be rare in brucellosis even in endemic area.* Brucella* involving different number of bursae (Table 1) have been reported. Previous reviewers have classified the olecranon bursa to be the most common site but our review of the recorded case revealed that prepatellar bursa is more common. This has been probably attributed to local trauma resulting from frequent kneeling among patients handling animals [5]. This patient might have also sustained repeated local trauma/pressure to the elbow as a result of the traditional use of cushion support at sheds by the Bedouin tribes of Arabia. The mode of infection in* Brucella* bursitis is believed to be due to haematogenous dissemination [7]. Bursitis can be the main manifestation of brucellosis. Fever and local signs of inflammation are absent in the majority of* Brucella* bursitis [5]. Most of* Brucella* bursitis patients have symptoms for long period ranging from few months to many years with the exception of few cases presented acutely. Diagnosis is usually but not always done by culturing the bursal aspirate. Standard tube agglutination (STA) test is traditionally used for serological detection of* Brucella* infection and has been reported to exhibit a sensitivity of 84.6%. The absence of serological evidence of* Brucella* infection in this patient could be due to failure to mount a detectable immune response that has been frequently observed among patients suffering from a localized disease [7].

*Brucella* species were isolated from the aspirate and the blood samples of the patient. The sensitivity of blood culture relies upon many factors including the stage of the disease and prior use of antimicrobials [9]. In acute cases, the sensitivities of the Ruz-Castaneda method and lysis centrifugation have been reported up to 80 and 90%, respectively, but among the chronic cases the sensitivities are 30 and 70%, respectively. The current case illustrates the lengthy delay in reaching a firm diagnosis in musculoskeletal brucellosis because of negative serology tests.

Treatment in the past favored surgical resection (bursectomy) but most experts perform treatment with antibiotics successfully. Combination of antibiotics for twelve weeks of therapy is advisable when focal disease is present. The patient improved on management with combination of streptomycin for two and doxycycline 100 mg twice daily for twelve weeks. This regime is very important as treatment failure and relapse rates occur from 6 to 24% for the oral regime as compared with 5 to 8% with oral/parenteral regime [10]. Patient has not shown evidence of relapse with follow-up.

## 4. Conclusion

This patient exhibited a rare form of osteoarticular complication of brucellosis. Furthermore the delay in reaching a firm diagnosis of brucellosis was due to the nonspecific clinical features and negative serology. This delay could lead to significant morbidity if blood and bursal fluid culture for brucellosis were not done.

## Figures and Tables

**Table 1:** Reported sites of bursal involvement.

Site	Number
Prepatellar	22
Olecranon	19
Subacromial	5
Suprapatellar	1
Iliopsoas	1
Subdeltoid	1
Greater trochanter	1
